# Advanced Imaging Methodology in Bacterial Biofilms with a Fluorescent Enzymatic Sensor for pepN Activity

**DOI:** 10.3390/bios14090424

**Published:** 2024-09-03

**Authors:** Javier Valverde-Pozo, Jose M. Paredes, María Eugenia García-Rubiño, María Dolores Girón, Rafael Salto, Jose M. Alvarez-Pez, Eva M. Talavera

**Affiliations:** 1 Nanoscopy-UGR Laboratory, Department of Physical Chemistry, Faculty of Pharmacy, Unidad de Excelencia en Quimica Aplicada a Biomedicina y Medioambiente (UEQ), University of Granada, C. U. Cartuja, 18071 Granada, Spain; javalverde@ugr.es (J.V.-P.); rubino@ugr.es (M.E.G.-R.); jalvarez@ugr.es (J.M.A.-P.); 2 Departamento de Química y Bioquímica, Facultad de Farmacia, Universidad San Pablo-CEU, CEU Universities, Urbanización Montepríncipe, 28668 Boadilla del Monte, Spain; 3 Department of Biochemistry and Molecular Biology II, Faculty of Pharmacy, Unidad de Excelencia en Quimica Aplicada a Biomedicina y Medioambiente (UEQ), University of Granada, Cartuja Campus, 18071 Granada, Spain; mgiron@ugr.es (M.D.G.); rsalto@ugr.es (R.S.)

**Keywords:** bacterial biofilms, pepN activity, bacterial processes, imaging techniques, fluorescence microscopy

## Abstract

This research explores the use of the pepN activity fluorescent sensor DCM-Ala in bacterial biofilms, emphasizing its significance due to the critical role of biofilms in various biological processes. Advanced imaging techniques were employed to visualize pepN activity, introducing a novel approach to examining biofilm maturity. We found that the overexpression of pepN increases the ability of *E. coli* to form biofilm. The findings demonstrate varying levels of pepN activity throughout biofilm development, suggesting potential applications in biofilm research and management. The results indicate that the fluorescent emission from this sensor could serve as a reliable indicator of biofilm maturity, and the imaging techniques developed could enhance our understanding and control of biofilm-related processes. This work highlights the importance of innovative methods in biofilm study and opens new avenues for utilizing chemical emissions in biofilm management.

## 1. Introduction

Bacterial biofilms are highly structured communities of bacterial cells (constituting 15% of their volume) encased in a self-produced extracellular polymeric matrix (comprising 85% of their volume). These biofilms adhere to both inert surfaces and living tissues [1,2] and represent the predominant mode of bacterial growth in nature. Biofilms can form under optimal conditions across a wide range of bacterial species. The matrix is primarily composed of water and various polymeric substances, with exopolysaccharides playing a central role [3,4]. These exopolysaccharides can be neutral/polyanionic (in gram-negative biofilms) or cationic (in gram-positive biofilms) [5,6]. In addition, the matrix contains proteins, DNA, RNA, lipids, and other biomolecules in smaller quantities [2,7,8]. Biofilms exhibit considerable variability in terms of thickness and size [9] and develop through distinct stages: initial attachment/adhesion, microcolony formation, biofilm matrix secretion, biofilm growth and maturation, and eventual detachment/release for colonization elsewhere [10]. Understanding bacterial biofilms is critical due to their significant impact on global health, with approximately 65% of bacterial infections attributed to biofilm formation [11]. These include infections such as native valve endocarditis, otitis media, osteomyelitis, and cystic fibrosis, as well as those associated with medical devices like central venous catheters and joint prostheses [12]. Biofilms are notably resistant to antimicrobial treatments (up to 1000 times more than isolated bacteria), contributing to antibiotic resistance and complicating eradication efforts. Their resilient structures are estimated to cause over 80% of chronic and recurrent microbial infections in humans [13]. Despite their adverse effects, biofilms also have beneficial applications, such as in probiotic delivery systems [14], driving research to understand their formation mechanisms and identify specific therapeutic targets [14,15,16,17].

Various methods, including electrochemical, optical, and mechanical tools, are currently employed to study biofilm properties [18]. The complexity of biofilms necessitates novel approaches that integrate direct imaging and sensing for detailed characterization. Fluorescence microscopy, known for its repeatability, non-invasiveness, and relative automation, is a prominent technique in biofilm studies, particularly for sensing pH [19], oxygen, heavy metals [20], and other factors [18,21]. Despite the availability of fluorescent sensors for bacterial enzymatic activity [22], research on enzymatic activity within biofilms remains scarce, with only a few examples documented in the literature [23,24,25]. Among bacterial enzymes, proteases are crucial in pathogenesis, with bacterial alanine aminopeptidase, encoded by the pepN gene in gram-negative bacteria like *E. coli*, being particularly significant. This enzyme could serve as a diagnostic marker for various gram-negative bacterial infections [26] and as a potential target for antimicrobial inhibitors (among which bestatin is notable) critical in addressing the challenges posed by rising antibiotic resistance [27].

In addition to imaging and measuring biofilms on surfaces, to obtain precise fluorescent images of enzyme distribution within tissues in vivo it is crucial to achieve good penetration of both excitation and emission. Short wavelengths are limited by autofluorescence, cell damage, and light scattering, challenges that can be mitigated by employing longer wavelengths in the red or near-infrared (NIR) range, which allows deeper penetration and reduces interference [28]. Two-photon excitation (TPE) is an effective strategy, where a molecule absorbs two photons simultaneously, each carrying half the excitation energy required for single-photon excitation [29]. TPE, first theoretically described by Maria Göppert-Meyer in 1931 [30], was widely adopted in fluorescence microscopy for biological studies in 1990, known as two-photon excitation fluorescence microscopy (TPM) [31]. TPM’s success is partly due to the use of NIR lasers, which reduce absorption and scattering in vivo compared to visible light [32], allowing for subcellular resolution at greater depths [33,34,35] while maintaining spatial resolution [36,37] and enhancing three-dimensional spatial localization [38]. As a result, TPM is gaining traction in clinical optical imaging [39].

Given the importance of bacterial biofilms in human health, this study employed single- and two-photon fluorescence microscopy to detect pepN activity during different stages of biofilm maturation. The DCM-Ala probe, a ratiometric fluorescent sensor with NIR emission, was used for this purpose [40]. Multiplexed measurements were performed using this sensor alongside blue fluorescent protein (BFP) as a bacterial body marker. Notably, the enzymatic reaction product—DCM-NH_2_ fluorophore—exhibits a high two-photon absorption cross-section (50 GM at 820 nm in DMSO [41]), making it suitable for bright imaging with a sufficient signal-to-noise ratio, even at low probe concentrations in TPM.

## 2. Materials and Methods

### 2.1. Reagents and Standards

All starting materials (reagents and solvents) were purchased commercially from Sigma-Aldrich (Madrid, Spain) with the highest degree of purity.

#### Sample Preparation and Experimental Measurements

The DCM-Ala and DCM-NH_2_ dyes were synthesized and purified as previously described [40]. The 0.5 mM stock solutions of both compounds were prepared in deuterated DMSO for purity testing by nuclear magnetic resonance.

Biofilms were prepared using 12-well cell culture plates, with each well containing 100 μL of XL1-Blue *E. coli* bacteria and 900 μL of LB growth medium (dilution 1/10). Bacteria were transformed with an empty expression vector (pMal-TEV-His) or an expression vector encoding for the pepN protease as described previously [40]. Growth of the transformed bacteria (induced or not with 1 mM IPTG) in minimal M9 media supplemented with glucose at 37 °C and shaking (200 rpm) was measured by absorbance at 600 nm. Colonies morphology was measured in LB agar plates, growth for 48 h at 37 °C.

For the analysis of biofilms, a square coverslip was inserted at a 45° angle, so that one part of the coverslip was fully submerged in the medium with the bacteria, while the other part remained outside the medium. In this setup, the samples were incubated for the necessary duration, depending on the specific day of biofilm activity to be analyzed. For acquisition of fluorescence images, the DCM-Ala substrate (5 μM) was deposited onto the coverslips with the formed biofilms. Subsequently, another coverslip was placed on top to ensure even distribution of the sensor across the entire sample.

### 2.2. Instrumentation

One-photon imaging was performed by a confocal Abberior microscope (Abberior Instruments GmbH, Heidelberg, Germany) using two pulsed excitation lasers (450 nm, 40 MHz and 375 nm, 40 MHz). The objective used was an UPlanSApo 100×/1.40 oil immersion device. The pinhole size was set to 1 AU. The collected fluorescence was separated by a 560LP dichroic directed to avalanche-photodiode (APD) and hybrid photomultiplier tube (HPMT) detectors after passing through 685/75 and 509/22 filters, respectively.

Two-photon imaging was performed using a confocal MicroTime 200 fluorescence microscope system (PicoQuant GmbH, Berlin, Germany). The excitation source was a Chameleon Discovery NX tunable laser (Coherent Laser Group, Santa Clara, CA, USA) used at an excitation wavelength of 800 nm. The repetition rate was modified by a pulse selector (APE Angewandte Physik & Elektronik GmbH, Berlin, Germany) using two acoustic–optic Bragg cells to reduce the frequency from 80 MHz to 40 MHz. The excitation beam passed through an achromatic quarter-wave filter (AQWP05-M-600, Thorlabs, Jessup, MD, USA) and was directed by an F73–705SG dichroic mirror (AHF/Chroma, Tübingen, Germany) to an inverted microscope system (IX-71, Olympus, Tokyo, Japan) with an oil immersion objective (1.4 NA, 100×). Fluorescence emission was collected with a 550 nm longpass filter (AHF/Chroma, Germany) and directed to a 150 μm pinhole. The emission from the sample was split into two detection channels after passing through a 600 DCXR dichroic beam splitter (AHF/Chroma), and then through bandpass filters, 685/70 (Semrock/AHF) until one detector and through 520/35 filter (Semrock/AHF) to the other detector. The detectors used were two different single-photon avalanche diodes (SPADs) (SPCM-AQR 14, PerkinElmer, Waltham, MA, USA).

Every image was exported as matrix data and analyzed using Fiji is just ImageJ. The analysis was performed taking channels separately. Ratiometric values between the red and green channels were calculated by homemade macros described in the SI and Figure S1.

## 3. Results and Discussion

### 3.1. Image Acquisition and Measurement Protocol

Bacterial biofilms represent intricate microbial ecosystems enveloped in extracellular polymeric substances. These biofilms can demonstrate heightened resilience to conventional antibiotics and instigate diseases via both device-associated and tissue-related infections, thereby posing a significant risk to global health. Consequently, the deployment of innovative methodologies to facilitate the study of biofilm development and the metabolic activities of bacteria during biofilm formation is crucial. This will enhance our comprehension of these biofilms and aid in the pursuit of novel therapeutic strategies [2].

**Figure 1:**
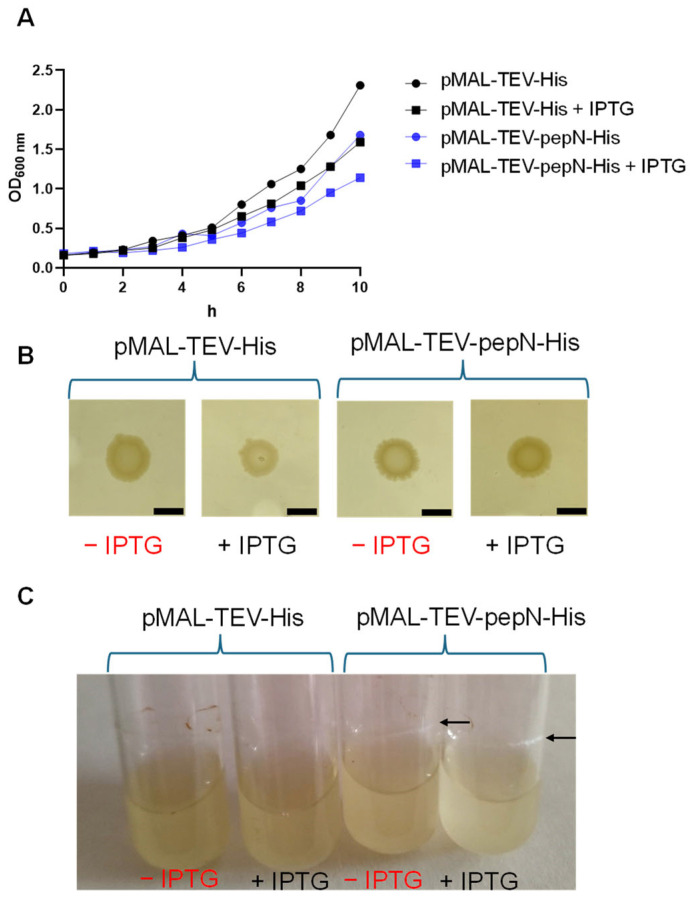
(**A**) Optical density of *E. coli* bacteria grown in M9 minimal medium supplemented with glucose. (**B**) Colonies of *E. coli* bacteria with an empty expression vector (pMAL-TEV-His) and a pepN vector (pMAL-TEV-pepN-His) grown in LB medium in the presence or absence of IPTG. Scale bars represent 10 mm. (**C**) *E. coli* bacteria with an empty vector (pMAL-TEV-His) and a pepN vector (pMAL-TEV-pepN-His) grown in M9 minimal medium supplemented with glucose. The arrows indicate the formation of biofilms in the tubes.

First, to better understand the role of pepN activity in the maturation cycle of biofilms, *E. coli* bacteria were transformed with either an empty expression plasmid or one containing the pepN sequence. The expression of the pepN protein was induced using Isopropyl β-D-1-thiogalactopyranoside (IPTG). In the absence of induction, there was moderate overproduction of pepN due to leakage from the promoter. When induced, the production of pepN was high. We found that pepN expression slows down the growth rate in a liquid medium (see Figure 1A). Moreover, colonies with pepN showed a fringed growth border (and an associated halo), which is typical of biofilm formation. In contrast, those with the empty vector had significantly less fringing (Figure 1B). While there are observable differences in biofilm morphology, the presence of the maltose-binding protein (MBP) in all plasmids leads to overexpression of MBP in the +IPTG bacteria. Although we did not find specific evidence in the literature to suggest a direct role for MBP in *E. coli* biofilm formation, we cannot completely rule out its potential significance. In other bacteria, such as *Cronobacter*, MBP has been shown to contribute to biofilm formation [42]. To corroborate that pepN activity can favor biofilm formation, we grew bacteria in a liquid medium and found that the production of biofilm was favored in the presence of this enzyme, as indicated by the white circles on the walls of the tubes (marked with arrows) containing bacteria with pepN in Figure 1C.

Our findings suggest that pepN plays a significant role in altering the growth dynamics of *E. coli* and enhancing biofilm formation. This provides new insights into the biological functions of pepN and its potential implications in bacterial growth and biofilm formation. Therefore, the use of sensors to detect pepN enzymatic activity is crucial in biofilm research, as it provides valuable insights into the metabolic processes within the biofilm, potentially unveiling new strategies for biofilm control and treatment.

**Figure 2:**
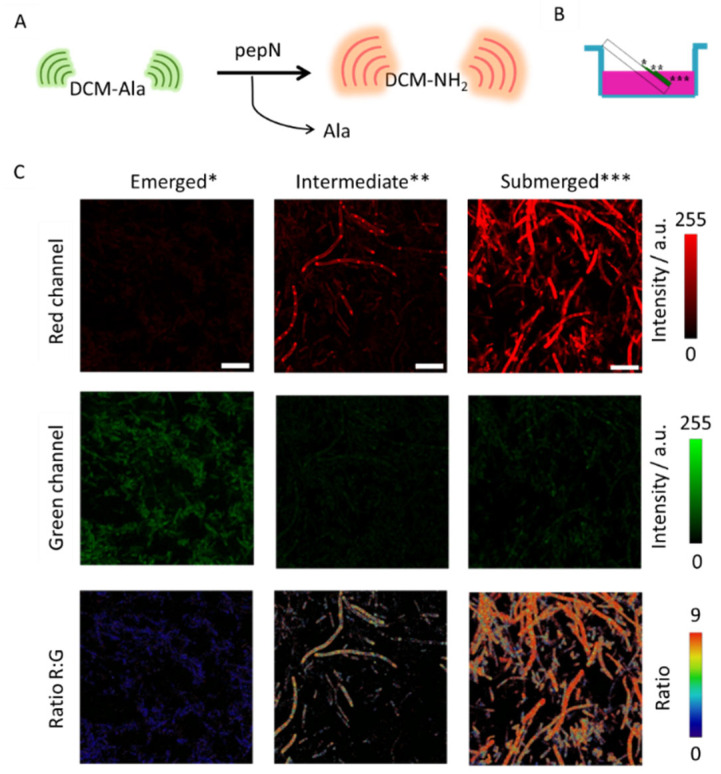
(**A**) Schematic representation of pepN releases DCM-NH_2_. (**B**) Illustration of the biofilm growth on the coverslip. *: Emerged region; **: Intermediate region; ***: Submerged region. (**C**) Representative images of the red (λ_em_ = 580–700 nm) and green (λ_em_ = 500–580 nm) intensity channels and the Ratio R:G images of 1-day maturated biofilm with induced pepN activity after 40 min of incubation with DCM-Ala (5 μM) by excitation at 450 nm. Scale bars are 10 μm.

In this study, we aim to utilize the fluorescent probe, DCM-Ala (chemical structure is presented in Scheme S1), to assess the activity of the enzyme pepN during the biofilm formation process in *E. coli*. The probe emits green fluorescence which transitions to red upon the hydrolysis of the peptide bond by the pepN enzyme, resulting in the removal of the alanine residue from the molecule releasing the compound DCM-NH_2_ (chemical structure is presented in Scheme S1) [40]. The ratio between the red and green fluorescence signals serves as an indicator of pepN activity (Figure 2A). Our initial approach was to ascertain our ability to detect varying enzymatic activity in distinct regions, both with and without biofilm formation. To establish regions with a gradient of biofilm formation, we cultivated bacteria on a slanted coverslip immersed in culture media (Figure 2B). This positioning enabled the development of a robust biofilm beneath the media, which gradually diminished as it reached the air interface.

After the biofilm had matured, we introduced the pepN sensor and measured the fluorescence intensity in both the red and green channels, as well as and the ratio between both channels (Ratio R:G) in the three regions after 40 min of incubation. Representative images of these regions are depicted in Figure 2C, revealing a significant difference among them. As can be observed, the emerged region shows a slight intensity in the red channel, while the intermediate region exhibits a substantial value, allowing for the identification of the hotspots previously observed in this bacterial body in our prior work [40]. Significantly, in the submerged region where the biofilm was more developed, there was a pronounced high value in red channel intensity. Of even greater significance than the increase in the red channel is the Ratio R:G value, as it determines enzymatic activity independently of probe concentration. These Ratio values confirm the difference in enzymatic activity among these three regions. As can be observed in Figure 2C, the ratio values suggest a notably higher alanine aminopeptidase activity in areas where the biofilm has grown more extensively. This experiment highlights the dependency of pepN activity on biofilm formation and confirms that the probe can be used in biologically complex structures as biofilms.

**Figure 3:**
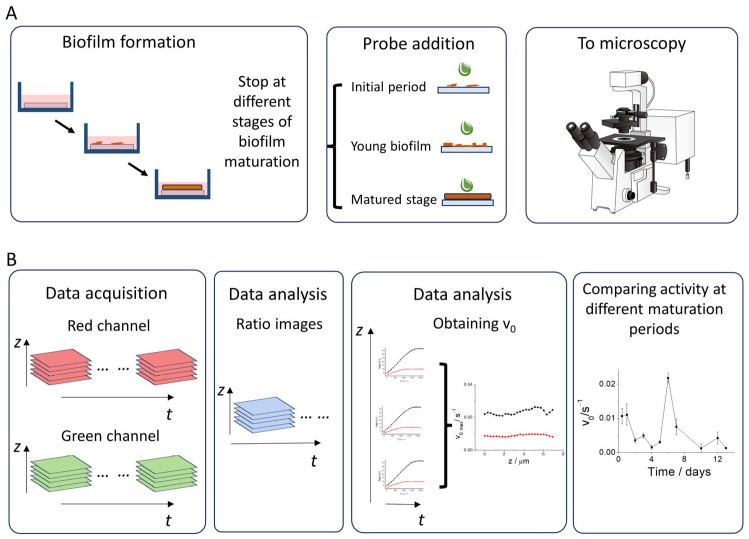
Schematic representation of the workflow performed in this study. The methodology is delineated into two main steps: (**A**) Biofilms sample preparation, (**B**) Imaging acquisition and analysis.

Upon confirming our ability to detect pepN activity in biofilms, and observing the intriguing results where the enzyme exhibits varying activity between biofilm formation and bacterial culture without biofilm formation, we focused toward enhancing the biological applications of this enzymatic fluorescent probe in biofilms, utilizing fluorescence microscopy. To achieve this, we devised the following work methodology, dividing the procedure into two distinct steps: (1) Biofilms sample preparation, (2) Imaging acquisition and analysis. The first step is illustrated in Figure 3A, where various biofilm samples were cultivated on coverslips immersed in culture media. These samples were collected at different maturation periods, and immediately before microscopy measurement, the pepN probe was introduced. The imaging acquisition and analysis is represented in Figure 3B. In summary, in the second step the fluorescence emission of the probe is detected across two distinct channels, and the images are acquired at various z-planes over time, thus enabling the generation of 3D images evolving temporally (first panel in Figure 3B). These images were used to obtain the ratio images using the Fiji is just ImageJ software (v 1.54f) [43] (second panel in Figure 3B), and the ratio values over time and in each z-plane are computed. Next, we represented the ratio values over time, obtaining a graphical representation for every z-plane (third panel in Figure 3B). In order to determine the reaction rate, we computed the derivative of the ratio values, which provided us with a measure of how the reaction rate changed over time. The first data point represents the initial rate (v_0_) of the enzymatic reaction. This rate is depicted over the depth of the biofilms (third panel in Figure 3B). The analysis, which involves calculating the initial rate in each z-plane, was conducted across a variety of biofilms with different levels of maturation, ranging from their inception to a maturation period of 13 days (fourth panel in Figure 3B).

### 3.2. Study of pepN Activity in Biofilms

Using the methodology proposed, we embarked on the initial step biofilm formation using *E. coli* bacteria culture. To procure biofilm samples, we introduced an aliquot of *E. coli* into the wells of a plate, each containing a coverslip. Following the 24 h incubation period, the biofilms began to form, and we were able to observe an initial biofilm on the coverslip surface. The coverslips remained in the well until the biofilms reached the desired level of maturation. Subsequently, we introduced the fluorescent pepN sensor, DCM-Ala, to the biofilm samples, and immediately proceeded with the image acquisition.

**Figure 4:**
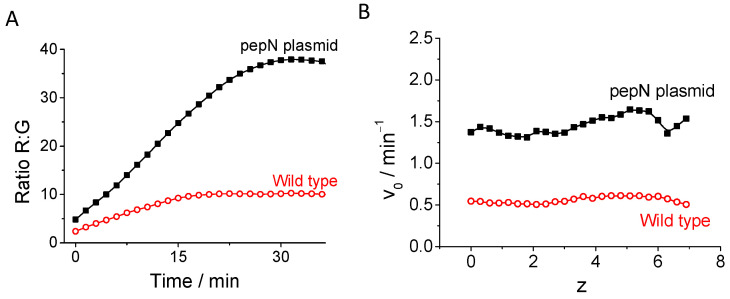
(**A**) Ratio R:G values of the middle plane of 6-day matured bacterial biofilms, comprising wild-type (open circles) and pepN plasmid bacteria (black squares) over 40 min. (**B**) Initial rates of 6-day matured bacterial biofilms from both populations at various z-planes.

Once the images are measured, the Ratio R:G is calculated in each plane through the analysis image software Fiji is just ImageJ [43]. Next, we select the bacterial bodies using an intensity threshold, the ratio value measured in each plane is plotted over time, and the slope of these curves is calculated to obtain the initial rate. An illustration of the increase in ratio values over time is provided in Figure 4A. The figure demonstrates the ratio values of two distinct bacterial populations: the wild-type, exhibiting a basal level of pepN activity, and the bacteria transformed with the pMAL-TEV-pepN-His plasmid, which display heightened expression of the pepN gene product [40]. The rate of ratio increase is contingent upon the enzymatic activity, determined by the slope value derived from the graphical representation of ratio values versus time. Our findings are consistent with the observation that the bacteria harboring the pMAL-TEV-pepN-His plasmid exhibit heightened pepN activity, as evidenced in Figure 4A. This analysis is conducted across every acquisition plane, and the rate of increase is depicted for each z-plane. We found, as illustrated in Figure 4B, that no significant variation in activity is observed across the different z-planes. Consequently, the bacteria demonstrate a consistent enzymatic activity throughout the entirety of the biofilm structure. The curves in every plane and the calculated rates are represented in Figure S2.

Next, we sought to decrease the enzymatic activity by using bestatin, an inhibitor of pepN activity [44], in wild-type bacterial biofilms that had matured for 6 days. As shown in Figure S3, biofilms incubated with bestatin exhibited a lower initial rate compared to the control biofilm. This reduction in the initial rate suggests that bestatin can effectively inhibit pepN activity within the biofilms, which may have significant implications for biofilm formation and development. These findings could pave the way for further studies into biofilm dynamics and the design of intervention strategies based on enzymatic inhibition. However, additional research is needed to fully explore these effects and determine their applicability in clinical or industrial contexts.

**Figure 5:**
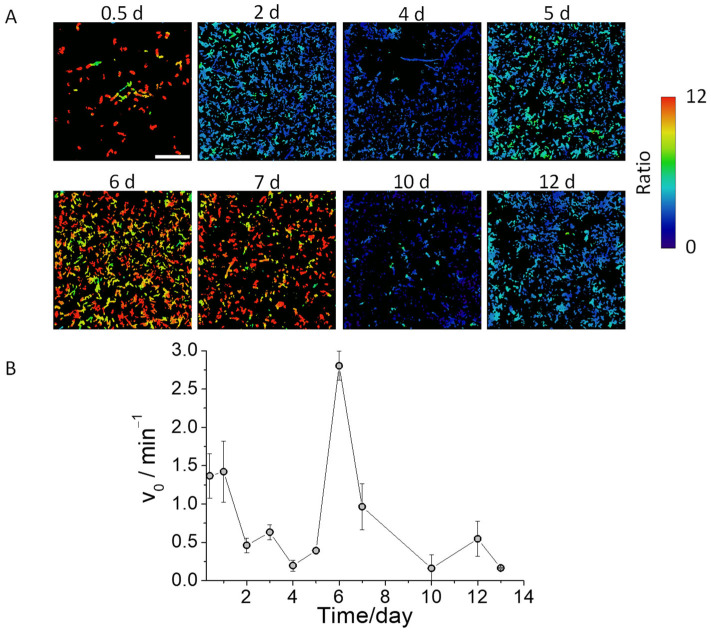
(**A**) Ratiometric images of *E. coli* biofilms with induced pepN activity from different days of maturation incubated with DCM-Ala (5 µm) for 15 min by excitation at 450 nm. Scale bar represents 20 μm. (**B**) Initial enzymatic reaction rates of *E. coli* biofilms with induced pepN activity from different days of maturation. Whiskers represent standard deviation.

We subsequently assessed pepN activity by quantifying the initial rate of fluorescence increase in induced pepN bacterial bodies across various stages of biofilm maturation, ranging from 0.5 to 13 days. In Figure S4, we present images captured using transmitted light microscopy, showcasing biofilms at various stages of maturation. This visual representation provides an overview of the biofilm’s developmental progression. Our findings reveal distinct levels of pepN activity at different stages of biofilm maturity. The obtained ratio values, which vary intriguingly, are represented in Figure 5A, which depicts images of the middle plane of bacterial bodies captured 15 min after the addition of DCM-Ala. The initial rates of fluorescence increase at different stages of biofilm maturity are calculated and illustrated in Figure 5B and in Figure S5. Based on these measurements, our observations highlight a decrease in pepN activity from the onset of the biofilm formation until the fourth or fifth day. Interestingly, this is followed by a significant surge on the sixth day of maturation. After this peak, there is a precipitous decrease in activity. This variability demonstrates that pepN activity is contingent upon the maturation stage of the biofilm. Similar results were obtained in bacteria with endogenous pepN activity, although, logically, the initial rate values were smaller due to the lower amount of enzyme present in the biofilm (see Figure S6).

The signals observed at 0.5 and 1 days can be attributed to the creation of initial *E. coli* aggregates, which are precursors to the formation of the matrix and exhibit appreciable pepN activity due to primary interactions between nearby cells. Subsequently, the bacteria begin to secrete hydrated extracellular polymeric substances, composed of polysaccharides, proteins, nucleic acids and lipids, beginning to form a three-dimensional network that interconnects and eventually immobilizes the bacteria in the biofilm [8]. For this reason, during the following 2 to 4 days, we observe a slight decrease in alanine aminopeptidase activity, which may result from the formation of a dense matrix that hinders probe diffusion, and reduces bacterial mobility and the process of cell-to-cell communication, leading to pepN secretion levels similar to those of isolated bacteria [45]. From days 5 to 7, the biofilms exhibit extensive aqueous channels within the bacterial network (Figure S3). The presence of these channels facilitates the diffusion of the probe, and the high local bacterial concentrations enhance cell-to-cell quorum sensing, leading to an increase in alanine aminopeptidase production. This results in a marked increase in the fluorescence signal in the red channel. Because the biofilm matrix also functions as an external digestive system by maintaining extracellular enzymes in the cellular surroundings (facilitating the metabolism of dissolved, colloidal, and solid biopolymers [8]), in older biofilms, the matrix metabolizes biopolymers, and bacteria become more individualized: similar to bacteria in solution, and exhibiting decreased pepN activity [46].

The application of this sensor has revealed different values in the pepN activity throughout the biofilm formation cycle. This approach provides the microbiologist with novel opportunities to investigate potential correlations between biofilm development and pepN activity. The experiments conducted validate the utility of this probe and methodology for evaluating pepN activity in complex biological samples, such as biofilms, a process of considerable significance in biomedical contexts. The implementation of this probe necessitates a ratio analysis based on emission wavelengths corresponding to the red and green regions of the electromagnetic spectrum. This ratiometric approach allows for more precise quantification by normalizing signal intensity, thereby reducing variability associated with fluctuations in marker concentration, illumination, and other experimental factors.

Examining this further, the real-time monitoring of pepN activity during biofilm formation could deepen our understanding of its role within the complex dynamics of biofilm communities. This idea may unveil new avenues for therapeutic interventions targeting biofilm-associated infections. Furthermore, employing ratio analysis based on emission wavelengths provides a robust and quantitative measure of pepN activity, essential for standardizing assessments of biofilm formation across different experimental setups or microbial strains. This methodology, therefore, represents a significant advancement in the field of microbiology and holds promise for future research in biofilm biology.

### 3.3. Multicolor Fluorescence Imaging and Two-Photon Excitation

This approach opens the door for the integration of a variety of spectrally compatible dyes into the probe, thereby enabling simultaneous measurement capabilities. To elaborate further, the ability to incorporate multiple dyes into the probe could revolutionize the field of biosensing. This could allow for the simultaneous detection and quantification of multiple targets within a single sample, enhancing the efficiency and comprehensiveness of the analysis.

**Figure 6:**
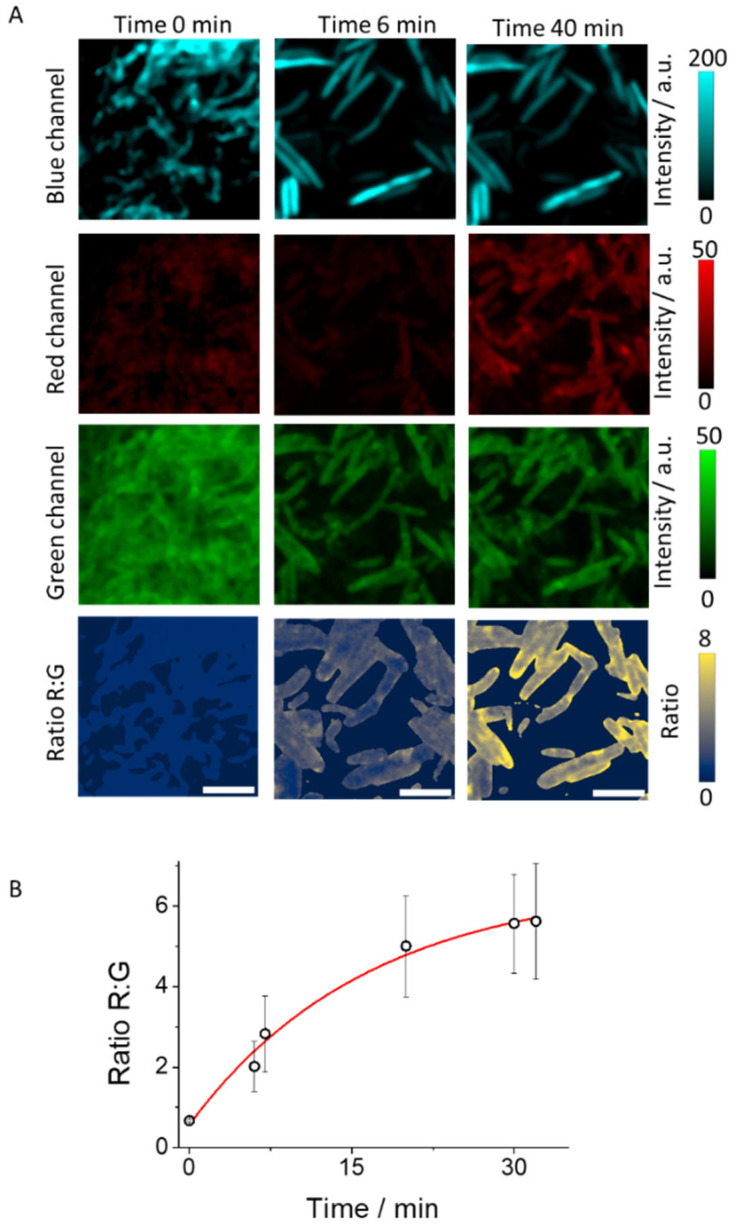
(**A**) Biofilm images with BFP at different incubation time of a 6-day maturation biofilm employing three detection channels, and the calculated Ratio R:G images. Scale bars represent 5 µm. (**B**) Average Ratio R:G values over time. Whiskers represent standard deviation.

To validate the ability to generate simultaneous images using different dyes, as a proof of concept we have incorporated blue fluorescent protein (BFP) into the bacterial bodies. This integration will expand our research by enabling us to observe bacterial bodies and analyze pepN activity under various spectral conditions. In this scenario, we capture fluorescence across three distinct channels: the blue channel to ascertain the location of the bacteria, the green channel to detect the fluorescence of the reactive DCM-Ala, and the red channel to capture the emission of the enzymatic reaction product, DCM-NH_2_. Figure 6A and Figure S7 show representative images from these three detection channels at different incubation times in a 6-day maturation biofilm.

As previously indicated, these data are used to calculate the ratio images. Figure 6B displays a plot of the ratio values of these bacterial bodies over time. The kinetics obtained are in accordance with the previous measurements. This multi-channel approach shows promising potential for advancing multi-analyte sensing, paving the way for new research avenues and applications in biotechnology and medicine. Moreover, the agreement in kinetics with previous measurements validates our methodology and emphasizes the reliability of our findings.

As previously indicated, bacterial biofilms produced by human pathogens are intricate structures that can be found on various human body tissues. Consequently, there is a significant interest in employing techniques that allow effective visualization in these structures. Currently, multiphoton excitation techniques are the most effective optical methods for visualizing the interior of biological bodies, owing to the relative transparency of organic tissues to IR radiation. DCM-NH_2_ has demonstrated robust absorption under two-photon excitation; therefore, in this research, we have utilized this methodology to visualize the bacterial biofilm.

**Figure 7:**
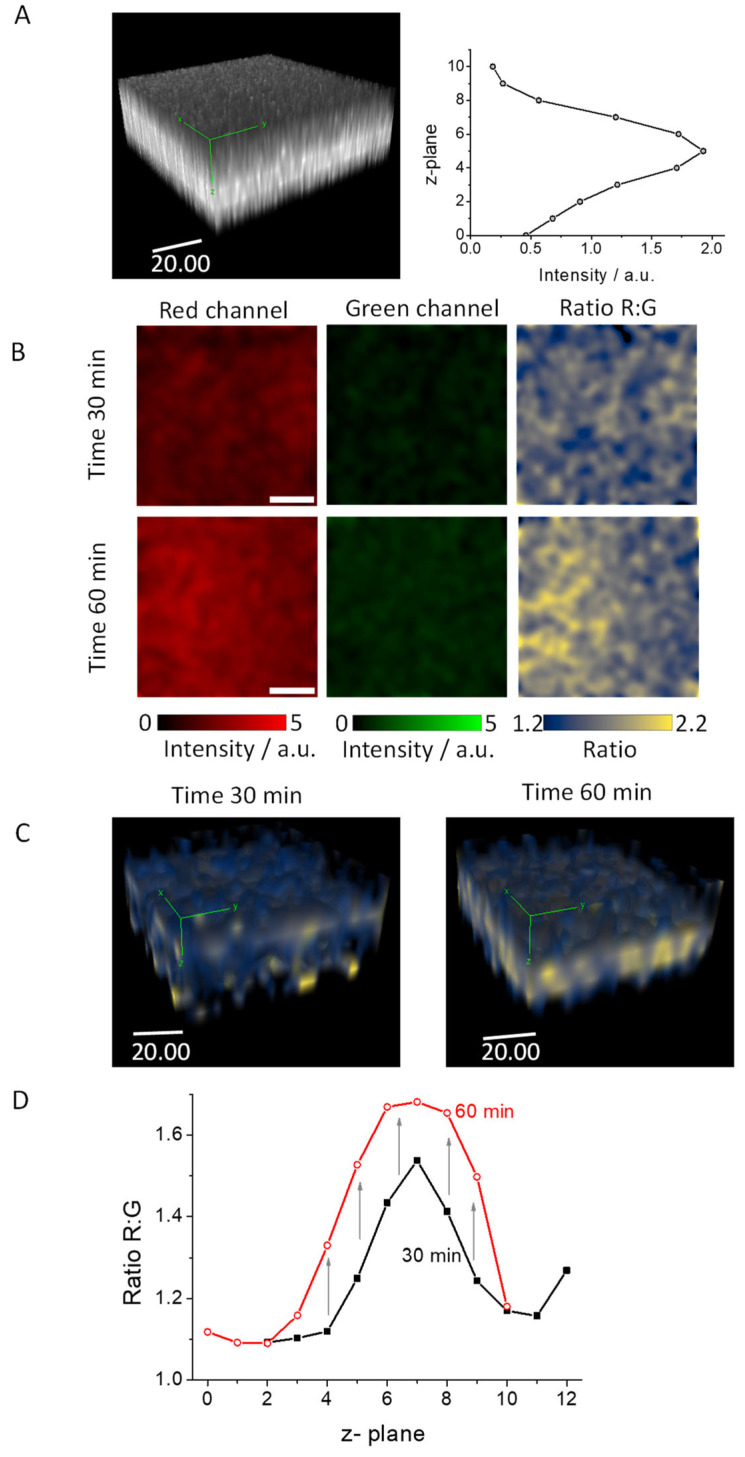
(**A**) Three-dimensional (3D) images of a 6-day mature biofilm after 30 min of incubation with DCM-Ala (10 µM), captured using two-photon microscopy with an excitation wavelength of 800 nm. Scale bar represents 20 μm. The right panel depicts the mean intensity values measured across each z-plane. (**B**) Red, green, and ratio images of the median plane of 6-day mature biofilms after 30 min (upper) and 60 min (lower) of incubation with DCM-Ala (10 µM). Scale bars are 20 μm. (**C**) 3D images of a 6-day mature biofilm after 30 min of incubation with DCM-Ala (10 µM), obtained using two-photon microscopy with excitation at 800 nm. (**D**) Ratio R:G values across each z-plane of a 6-day mature biofilm following 30 and 60 min of incubation with DCM-Ala (10 µM). Scale bars represent 20 μm. The green lines in panels (**A**) and (**C**) serve to delineate three-dimensional visualization.

As depicted in Figure 7, our results demonstrate a strong emission under two-photon excitation conditions across the entire thickness of a mature biofilm. Figure 7A represents the center of the biofilm, while Figure S8 illustrates the edge of this structure. The right panels in these figures display the calculated mean intensity values at each measured z-plane. As can be discerned from these panels, a strong signal corresponds to the presence of DCM-NH_2_ emission in the biofilms after 40 min of incubation with DCM-Ala.

To harness the advantages of two-photon excitation, we collected the fluorescence signal, which was divided into two distinct channels (red and green) across different z-planes. Subsequently, we computed the Ratio R:G images. In Figure 7B, we present the images from the median plane of these channels after 30 and 60 min of incubation with DCM-Ala. As can be observed, there is a noticeable increase in the red channel, leading to a corresponding rise in the R:G ratio images. In contrast, the green channel presents a less fluorescent signal which could be due to the lower two-photon absorption cross-section of DCM-Ala, caused by the coupling of the amino acid alanine to the fluorophore [40].

In Figure 7C, we display the 3D ratio images, where the increase, primarily noticeable in the biofilm’s center, can be visualized after 60 min. Lastly, we quantified the intensity values of the ratio for both channels across all z-planes. These values are illustrated in Figure 7D, where a general increase across all the z-planes is observed. This change signifies that incubation with DCM-Ala leads to a significant change in the fluorescence signal, particularly in the red channel, indicating detection of enzymatic activity in the biofilm. The entirety of the biofilm’s structure can be monitored through the ratio value using the DCM-Ala probe. This methodology paves the way for new opportunities in studying enzymatic activity within biofilms located inside tissues or materials. It provides a novel approach to understanding the complex dynamics of biofilms’ behavior and their interactions with their surrounding environment. This can lead to the development of more effective strategies for managing biofilm-related issues in various fields, including healthcare and materials science.

It is noteworthy that the strong emission of DCM-NH_2_ in the biofilms can serve as a reliable indicator of biofilm maturity, which opens up new avenues for further research into the dynamics of biofilm development and the potential implications for bacterial pathogenicity. Future studies will be able to utilize the correlation between the intensity of DCM-NH_2_ emission and specific stages of biofilm development, providing a deeper understanding of the life cycle of bacterial biofilms. This could have significant implications for the development of strategies to control biofilm formation in various contexts, from medical to industrial applications. 

## 4. Conclusions

In this study, we investigated the enzymatic activity of pepN during biofilm formation using the fluorescent sensor DCM-Ala. Our findings revealed distinct activity patterns throughout the biofilm maturation process, with a notable peak around 6 days. Additionally, we explored the use of multiplexing by incorporating BFP as a marker of bacterial bodies within the biofilm matrix. Finally, we successfully confirmed the applicability of the DCM-Ala probe in biofilms using TPE.

The detection of pepN enzymatic activity using DCM-Ala highlights its potential as a powerful tool for studying biofilm dynamics. Future studies should be conducted to explore additional applications of DCM-Ala and optimize its use in biofilm research.

## Data Availability

Data will be made available on request.

## Supplementary Materials

The following supporting information can be downloaded at: https://www.mdpi.com/article/10.3390/bios14090424/s1, Image analysis and macros. Figure S1. Steps of Image Analysis Conducted. Scheme S1. Chemical structures of the DCM-Ala probe and the DCM-NH_2_ compound and proposed hydrolysis reaction. Figure S2. Kinetic curves and rate values. Figure S3. Representation of the R:G ratios without (circles) and with (squares) bestatin. Figure S4. Transmitted images of biofilms at different stages of biofilm maturation. Figure S5. Representative 3D-ratio images of biofilms at different times. Figure S6. Initial enzymatic reaction rates of *E. coli* biofilms with endogenous pepN activity from different days of maturation. Figure S7. Biofilm images with BFP at different incubation times of a 6-day maturation biofilm employing three detection channels, and the calculated Ratio R:G images. Figure S8. 3D image of edge of the biofilm excited by TPE.
