# Supplementary material for: Advanced Imaging Methodology in Bacterial Biofilms with a Fluorescent Enzymatic Sensor for pepN Activity
Source: Biosensors (Basel). 2024 Sep 3;14(9):424. doi: 10.3390/bios14090424 (PMC11430670; doi:10.3390/bios14090424)

Supporting Information

Advanced Imaging Methodology in Bacterial Biofilms with a Fluorescent Enzymatic Sensor for pepN Activity

Javier Valverde-Pozo [1,2], Jose M. Paredes [1,*], M. Eugenia Garcia-Rubiño [1], Maria D. Giron [3], Rafael Salto [3], Jose M. Alvarez-Pez [1] and Eva M. Talavera [1,*]

[1] Nanoscopy-UGR Laboratory, Department of Physical Chemistry, Faculty of Pharmacy, Unidad de Excelencia en Quimica Aplicada a Biomedicina y Medioambiente (UEQ), University of Granada, C. U. Cartuja, 18071 Granada, Spain

[2] Departamento de Química y Bioquímica, Facultad de Farmacia, Universidad San Pablo-CEU, CEU Universities, Urbanización Montepríncipe, 28668, Boadilla del Monte, Spain

[3] Department of Biochemistry and Molecular Biology II, Faculty of Pharmacy, Unidad de Excelencia en Quimica Aplicada a Biomedicina y Medioambiente (UEQ), University of Granada, Cartuja Campus, 18071, Granada, Spain

* Correspondence: EMT: etalaver@ugr.es; JMP: jmparedes@ugr.es

	Page
Image Analysis and macros.	2
**Figure S1.** Steps of Image Analysis Conducted.	2
**Scheme S1.** Chemical structures of the DCM-Ala probe and the DCM-NH_2 compound and proposed hydrolysis reaction.	3
**Figure S2.** Kinetic curves and rate values.	4
**Figure S3.** Representation of the R:G ratios without and with bestatin.	5
**Figure S4.** Transmitted images of biofilms at different stages of biofilm maturation.	6
**Figure S5.** Representative 3D-ratio images of biofilms at different times.	7
**Figure S6.** Initial enzymatic reaction rates of *E. coli* biofilms with endogenous pepN activity from different days of maturation.	8
**Figure S7.** Biofilm images with BFP at different incubation time of a 6-day maturation biofilm employing three detection channels and the calculated Ratio R:G images.	9
**Figure S8.** 3D image of edge of the biofilm excited by TPE.	10

Image analysis and macros.

Each image was exported as matrix data and analyzed using the software "Fiji is just ImageJ". The channels measured were labeled as blue (for the BFP, although not all experiments utilized this channel), green (for the detection of DCM-Ala emission), and red (for the detection of DCM-HN_2 emission). Ratiometric values between the red and green channels were calculated using custom macros, following these steps:

1. Initially, the images from all channels were converted into 32-bit format. Subsequently, Gaussian blur filters (σ=1) were applied to each channel.
2. For the selection of bacterial bodies, we duplicated the red or blue channel (the latter only in experiments using BFP) to generate the ROI (Region of Interest) channel. A Gaussian blur filter (σ=2) was then applied to this channel. Bacterial bodies were selected using an intensity threshold, and pixels falling outside this threshold were converted to NaN (Not a Number).
3. ROI was applied to the red and green channels.
4. Red channel was divided by the green channel to obtain the ratio images.

Figure S1. Steps of Image Analysis Conducted.

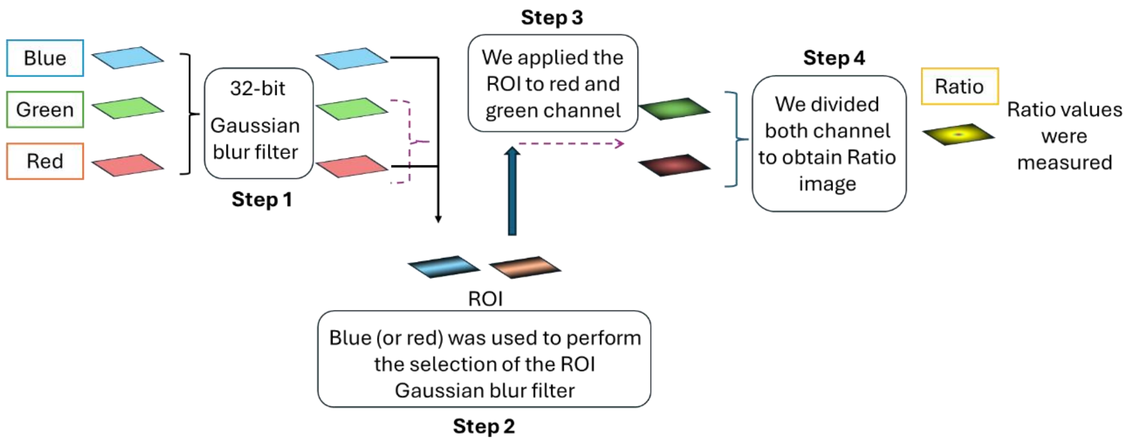

Scheme S1. Chemical structures of the DCM-Ala probe and the DCM-NH_2 compound and proposed hydrolysis reaction.

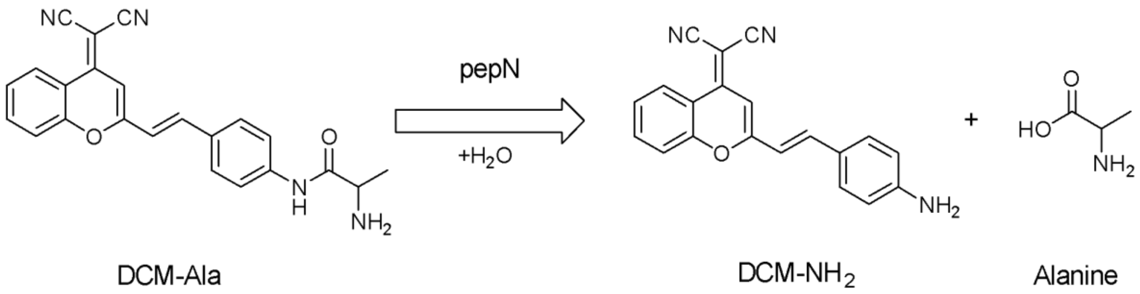

Figure S2. Kinetic curves and rate values.

Day | Kinetics in different z-planes | Kinetics in the middle plane | Rate

1

3

6

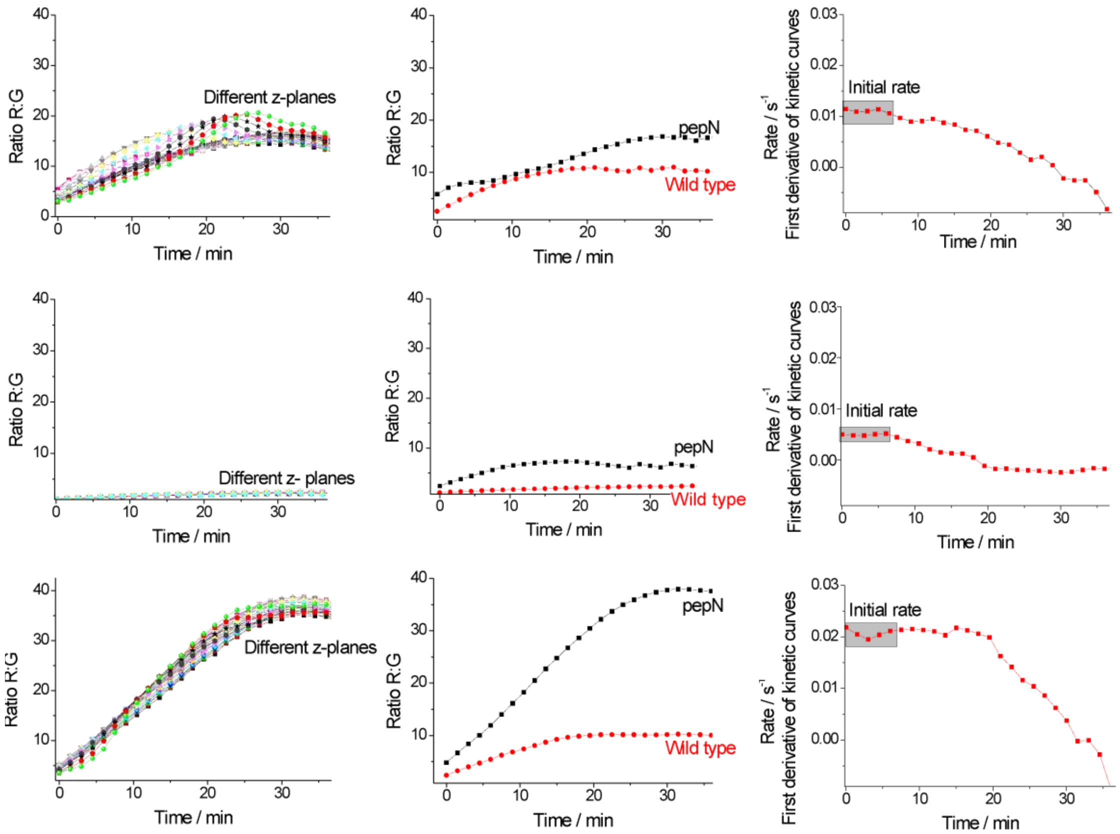

Figure S3. Representation of the R:G ratios without (circles) and with (squares) bestatin in 6-days matured biofilms.

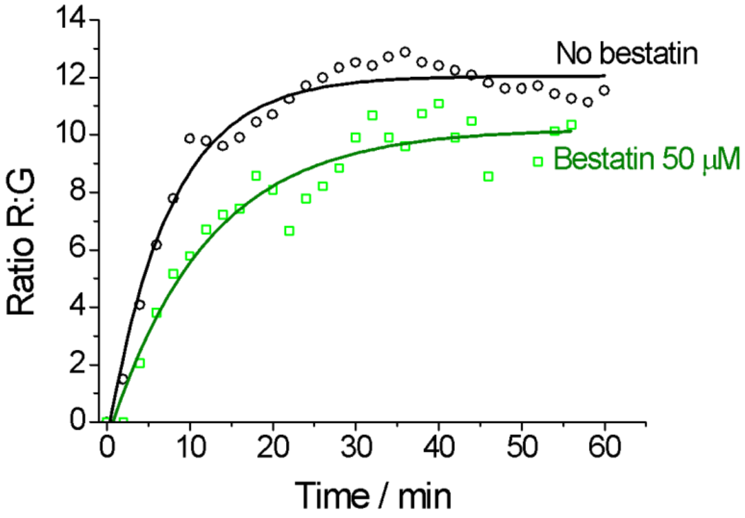

Figure S4. Transmitted images of biofilms at different stages of biofilm maturation.

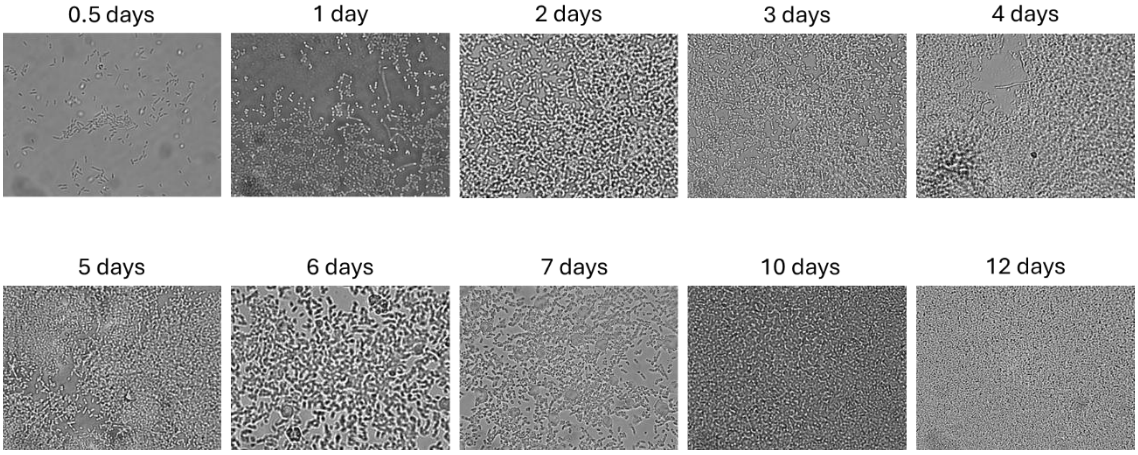

Figure S5. Representative 3D-ratio images of biofilms at different times.

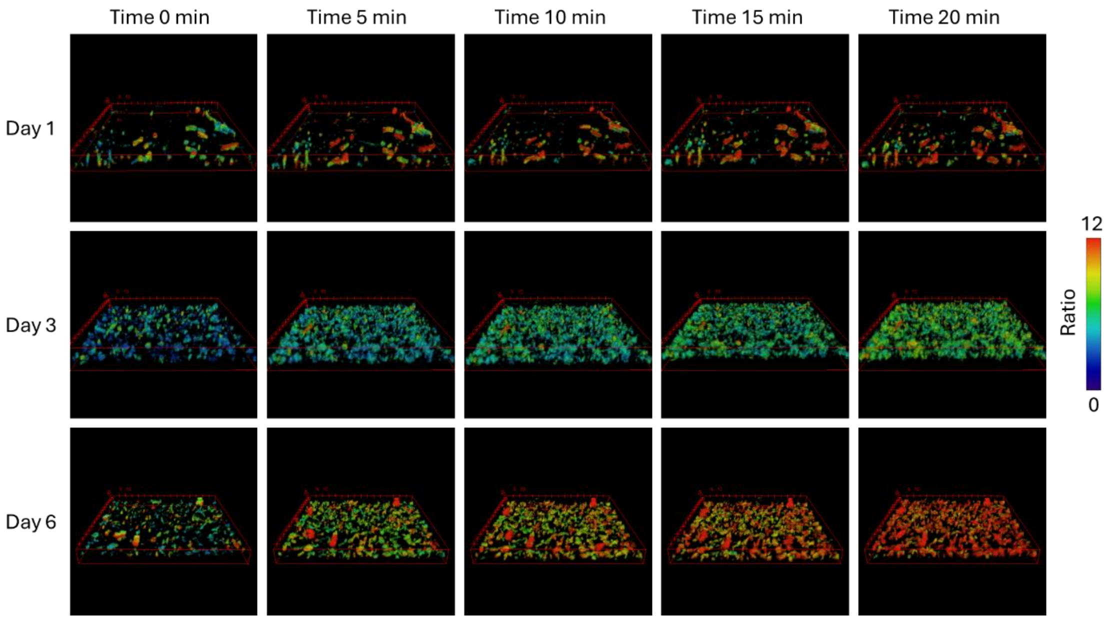

Figure S6. Initial enzymatic reaction rates of *E. coli* biofilms with endogenous pepN activity from different days of maturation.

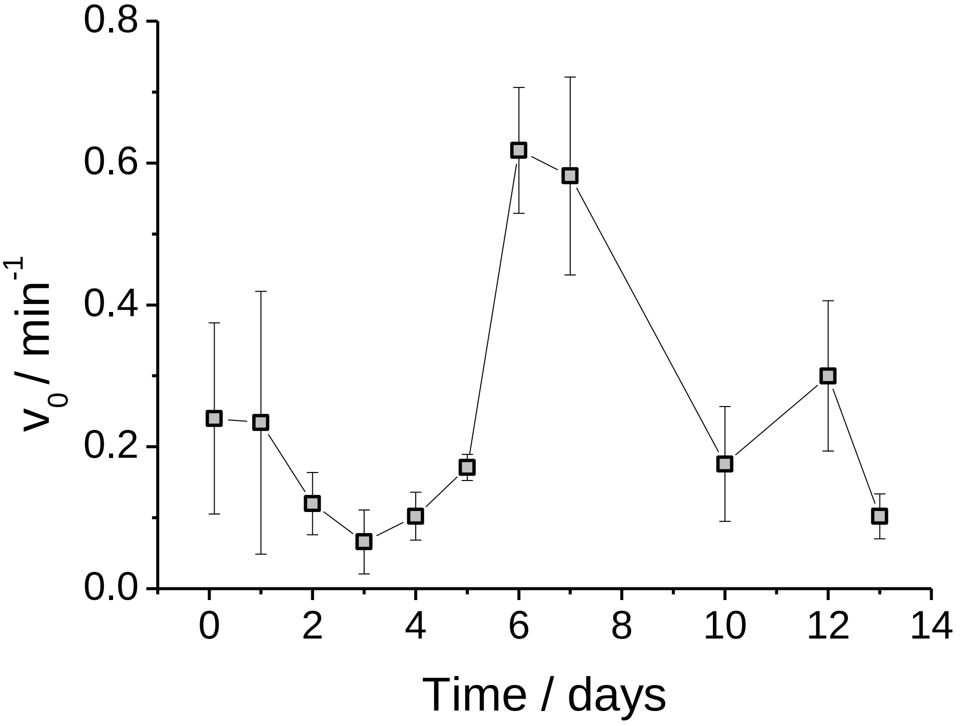

Figure S7. Biofilm images with BFP at different incubation time of a 6-day maturation biofilm employing three detection channels and the calculated Ratio R:G images. Scale bars represent 2 µm.

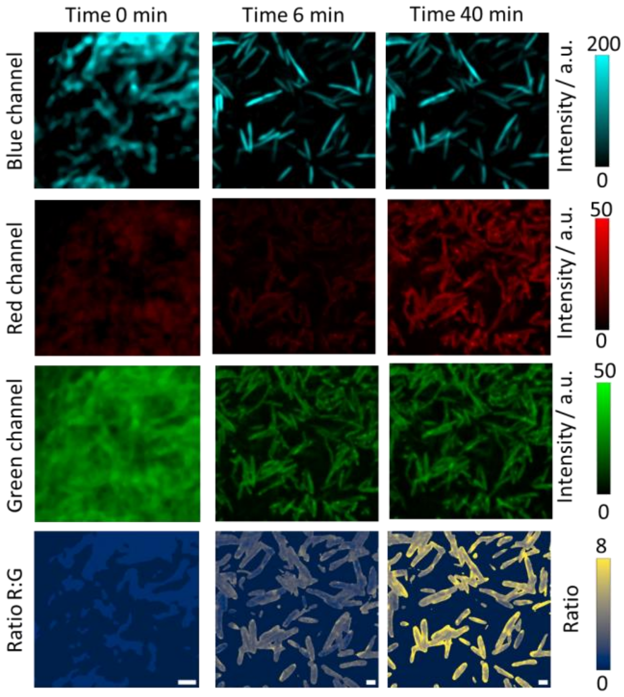

Figure S8. 3D image of edge of the biofilm of a 13-day mature biofilm after 30 minutes of incubation with DCM-NH-Ala (10 μM) using two-photon microscopy with excitation at 800 nm. Right panel represents the mean intensity values in every z-plane measured.

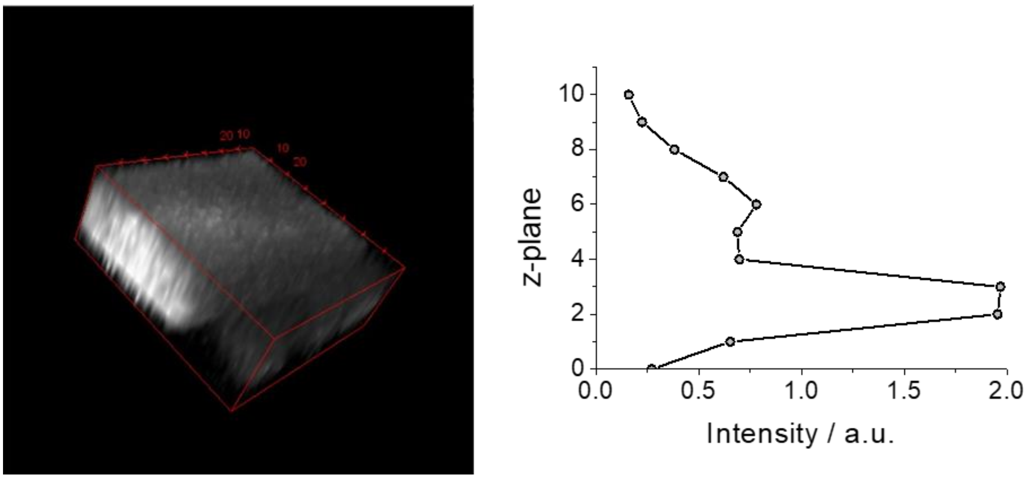

Supplement: Supplementary file 1 [file biosensors-14-00424-s001.zip › biosensors-3143580-supplementary.pdf]
